# Two C3H Type Zinc Finger Protein Genes, *CpCZF1* and *CpCZF2*, from *Chimonanthus praecox* Affect Stamen Development in *Arabidopsis*

**DOI:** 10.3390/genes8080199

**Published:** 2017-08-10

**Authors:** Huamin Liu, Renwei Huang, Jing Ma, Shunzhao Sui, Yulong Guo, Daofeng Liu, Zhineng Li, Yechun Lin, Mingyang Li

**Affiliations:** 1 Chongqing Engineering Research Center for Floriculture, Key Laboratory of Horticulture Science for Southern Mountainous Regions, Ministry of Education, College of Horticulture and Landscape, Southwest University, Chongqing 400715, China; liuhuanin@126.com (H.L.); swuhrw@126.com (R.H.); majing427@swu.edu.cn (J.M.); sszcq@swu.edu.cn (S.S.); yulong@swu.edu.cn (Y.G.); liu19830222@163.com (D.L.); znli@swu.edu.cn (Z.L.); 2 Upland Flue-Cured Tobacco Quality and Ecology Key Laboratory of China Tobacco, Guizhou Academy of Tobacco Science, Guiyang 550003, China; linyechun@live.cn

**Keywords:** C3H-type zinc finger protein, CpCZF1 and CpCZF2, over-expression, flower development

## Abstract

Wintersweet (*Chimonanthus praecox*) is a popular garden plant because of its flowering time, sweet fragrance, and ornamental value. However, research into the molecular mechanism that regulates flower development in wintersweet is still limited. In this study, we sought to investigate the molecular characteristics, expression patterns, and potential functions of two C3H-type zinc finger (CZF) protein genes, *CpCZF1* and *CpCZF2*, which were isolated from the wintersweet flowers based on the flower developmental transcriptome database. *CpCZF1* and *CpCZF2* were more highly expressed in flower organs than in vegetative tissues, and during the flower development, their expression profiles were associated with flower primordial differentiation, especially that of petal and stamen primordial differentiation. Overexpression of either *CpCZF1* or *CpCZF2* caused alterations on stamens in transgenic *Arabidopsis*. The expression levels of the stamen identity-related genes, such as *AGAMOUS* (*AG*), *PISTILLATA* (*PI*), *SEPALLATA1* (*SEP1*), *SEPALLATA2* (*SEP2*), *SEPALLATA3* (*SEP3*), *APETALA1* (*AP1*), *APETALA2* (*AP2*), and boundary gene *RABBIT EAR* (*RBE*) were significantly up-regulated in *CpCZF1* overexpression lines. Additionally, the transcripts of *AG*, *PI*, *APETALA3* *SEP1-3*, *AP1*, and *RBE* were markedly increased in *CpCZF2* overexpressed plant inflorescences. Moreover, CpCZF1 and CpCZF2 could interact with each other by using yeast two-hybrid and bimolecular fluorescence complementation assays. Our results suggest that CpCZF1 and CpCZF2 may be involved in the regulation of stamen development and cause the formation of abnormal flowers in transgenic *Arabidopsis* plants.

## 1. Introduction

Flowering plants are one of the most diverse groups of organisms on earth, constituting 295,383 of the 374,000 known plant species in the wild [1]. So far, the genes governing flower development and organ identities have been extensively studied in various plants. As a model plant, the flower of *Arabidopsis* has four whorls of organs. The A-, B-, C-, D-, and E-function of the floral homeotic genes were found to be vital for flora primordial identity specification [2,3,4,5,6,7,8], and constitute the most regulators of the flower organ identities. Most of the regulators are transcriptional factors, where *APETALA2* (*AP2*) encodes an AP2/EREBP transcriptional factor protein [9], and the rest of the A-, B-, C-, D-, and E-function genes code for MADS-box domain transcriptional factor proteins [10]. Moreover, members of other transcriptional factor families have been identified for their role in directly or indirectly regulating flower development. For example, the flowering time in maize is controlled by an Ap2-like gene (*ZmRap2.7*) orthologous to Rap2.7, a transcription factor that regulates the flowering time in *Arabidopsis* [11]. S*PATULA* (*SPT*), a basic helix-loop-helix (bHLH) type transcriptional factor, controls carpel development in *Arabidopsis* [12,13]. HUA1, a zinc finger protein with six CCCH motifs, indirectly determines carpel and stamen identity specification by acting in the processing of *AGAMOUS* (*AG*) Pre-mRNA in a certain genetic background [14,15,16]. In plants, zinc finger proteins (ZFPs) are among the most abundant proteins, and they were reported to be involved in various activities of plant growth, development, phytohormone signaling, and stress responses [17]. According to the number and order of conserved cysteine and histone residues, ZFPs were classified into several distinct types, such as C2H2, C3H, C2H2, C3HC4, C2HC5, and C3H2C3 [18]. Among them, the C3H-type zinc finger proteins are characterized by a typical motif consisting of three cysteine residues and one histidine residue; they usually contain 1–6 C-X_6-14_-C-X_4-5_-C-X_3_-H motifs, wherein the X is any amino acid [19], with the exception of ZmC3H3 from *Zea mays*, which contains seven CCCH motifs [20]. In recent years, a small number of C3H-type genes have been identified and functionally studied, which were known to function in various life processes in plants. OsLIC, a C3H-type zinc finger protein with a single CCCH zinc finger motif, acts as a negative regulator of the leaf and tiller angle in rice through mediating the brassinosteroids responses [21]. OsDOS and OsTZF1 were found to delay the leaf senescence in rice [22,23]. *CsSEF1*, a tandem C3H-type zinc finger protein gene from cucumber, plays a role in the signal transduction pathway from the photoassimilate limitation to growth cessation [24]. *AtTZF4*/*SOMNUS*, *AtTZF*5, and *AtTZF*6*/PEI1* all code for proteins with a tandem CCCH zinc finger motif, and are involved in the light-, abscisic acid-, and gibberellic acid- mediated regulation of seed germination [25,26]. AtSZF1 and AtSZF2 are involved in salt stress responses in *Arabidopsis* and their mutants display more sensitive responses to salt stress than wild-type plants [27]. Moreover, AtC3H14 and AtC3H15 have overlapping roles in the regulation of second wall thickening and flower development [28,29]. GhZFP1, a novel C3H-type zinc finger protein from cotton, enhances salt stress tolerance and fungal disease resistance in tobacco [30]. MsZFN from alfalfa delays the flowering time by repressing the flowering genes in transgenic *Arabidopsis* [31].

Wintersweet (*Chimonanthus praecox*), a basal angiosperm belongs to the Calycanthaceae family, is a precious shrub endemic to China. The wintersweet flowers are characterized by a strong fragrance and blossom in winter, from November to March. Its prized ornamental value, unique flowering time, and attractive sweet scent make it an admired landscape plant or cut flower plant in China. There are several traits and properties of wintersweet that are important in a commercial context, including flower development, senescence, scent biosynthesis and emission, and resistance to abiotic stresses [32,33]. Researches have mainly focused on the identification of abiotic stress responsive genes [32,34] and analysis of essential oil in wintersweet [35]. To date, however, only two genes involved in flower organ identity have been reported. *CpAP3*, a B-function gene, caused rich alterations in the petals and stamens of petunia, partially rescuing the stamen development and fertility in the *Arabidopsis ap3* mutant; *CpAGL6*, a *SEP1*-like gene, regulated the stamen and carpel identities and caused partial sterility in transgenic *Arabidopsis.* Nevertheless, the molecular mechanism regulating flower organ identity in wintersweet is still largely unknown [36]. 

A former transcriptomic analysis of flower development in wintersweet revealed that a large number of candidate genes expressed differentially during flower development [33]. Among them, some C3H-type zinc finger protein genes, such as *CpCZF1* and *CpCZF2*, were found to be more highly expressed in the flower bud stage and less expressed in the senescence stage. In this study, the results show that these two C3H-type Zinc Finger genes, *CpCZF1* and *CpCZF2*, isolated from wintersweet, may be involved in the regulation of flower organ identity in transgenic *Arabidopsis*. Our study thus provides useful knowledge for better understanding the regulation of flower development and the functional roles of C3H-type zinc finger proteins in plants.

## 2. Materials and Methods

### 2.1. Plants and Growing Conditions

For the dynamic expression assays in flower development, flower buds at four primordial differentiation stages and whole flowers at six flowering stages were harvested. The former were associated with sepal primordia differentiation in early April, followed by that of the petal in late April, the stamen in early May, and the pistil in early July [37]. Following Ma et al. [38], flowering stage separation occured as follows: Stage 1 (flower-bud), Stage 2 (petal-display), Stage 3 (initiating bloom), Stage 4 (bloom), Stage 5 (early-withering), and Stage 6 (late-withering). For gene expression analysis in tissues, the wintersweet seeds were treated with 70% sulfuric acid for 30 minutes and cleaned before being sown directly into pots containing a mixture of substrate and vermiculite (3:1, *v*/*v*); the wintersweet adults were planted in the nursery at Southwest University (Chongqing, China). The cotyledons were harvested when they emerged from beneath the seed coat; the root, stem, and young leaves were harvested from the four-leaf stage plants. The mature leaves, and petals, stamens, and pistils from the flowers at stage1 were harvested from adult plants. 

For the subcellular localization and bimolecular fluorescence complementation (BIFC) analyses [39], tobacco (*Nicotiana benthamiana*) seeds were sown directly into pots containing a mixture of substrate and vermiculite (3:1, *v*/*v*) and grown under a 16-h light (28 °C)/8-h dark (22 °C) photoperiod. 

For plant transformation, the *Arabidopsis thaliana* seeds were sown on Murashige and Skoog (MS) solid medium containing 3% (*w*/*v*) sucrose and 0.7% (*w*/*v*) agar, stratified for three days at 4 °C, and then transferred to a growth room at 70% relative humidity under a 16-h light (22 °C)/8-h dark (20 °C) photoperiod and 120 µmol·m^−2^·s^−1^of cool white fluorescent light. For those *Arabidopsis* plants used in the phenotypic analysis, 12-day-old seedlings were transplanted into plastic pots containing a mixture of substrate and vermiculite (3:1, *v*/*v*) under the same controlled environmental conditions.

### 2.2. Cloning and Sequence Analysis

Total RNA was extracted from the flowers of wintersweet by using the RNAprep pure kit (Tiangen Biotech, Beijing, China). This RNA sample was used to synthesize the cDNA sample, which was then used to perform 5′ rapid amplification cDNA end (RACE) following the protocol of the SMARTer RACE 5′/3′ Kit (Clontech, CA, USA). Primers for 5′RACE were designed using the Primer 5 Program, as based on the sequence which encoded the 3′ ends of putative ‘C3H’ genes obtained from the transcriptome database of flower development [33]. The 5′RACE PCR procedure consisted of: five cycles at 94 °C for 30 s, 72 °C for 3 min; five cycles at 94 °C for 30 s, 70 °C for 30 s, 72 °C for 3 min; 25 cycles at 94 °C for 30 s, 68 °C for 30 s, and 72 °C for 3 min. Full-length cDNA of these two putative ‘C3H’ genes were generated using PCR with the specific primers (Supplementary Materials Table S1). The PCR products were cloned into the pMD19-T Easy vector (Takara, Dalian, China) and sequenced. The full-length cDNA was analyzed by the BLAST program at the National Center for Biotechnology Information (NCBI) website (http://www.ncbi.nlm.nih.gov/). Multiple Sequence Alignment was carried out using the online tool MSA (http://www.ebi.ac.uk/Tools/msa/). A phylogenetic tree was constructed using Molecular Evolutionary Genetics analysis version 6.0 (http://www.megasoftware.net/) based on the neighbor joining method. 

### 2.3. Subcellular Localization 

The coding sequences of *CpCZF1* and *CpCZF2* were cloned into the modified pCAMBIA 1300 vector (*35S* promoter, C-*GFP*), respectively, at the *BamH*I and *Sai*I, *Sac*I, and *BamH*I sites. The primers for this construction are listed in Supplementary Materials Table S1. *Agrobacterium tumefaciens* GV3101 was transformed by the control vectors *35S:GFP* (i.e., the modified pCAMBIA 1300), and the *35S:CpCZF1-GFP* and *35S:CpCZF2-GFP* constructs were used to separately infiltrate the underside of the top leaves of the tobacco plants*.* After incubation in the dark at 22 °C for 36 h, a 2-mm × 2-mm area portion of the infiltrated leaves was cut out and incubated in distilled water supplemented with 5 µg/mL 4′,6-diamidino-2-phenylindole (DAPI; Sigma, St. Louis, MO, USA) for 30 min [40]. Afterwards, leaf sections were mounted on a microscope slide and covered with distilled water for observing the green fluorescent protein via confocal microscopy (Olympus, FV-10-ASW, Tokyo, Japan).

### 2.4. Quantitative Real Time-PCR

Total RNA was extracted by using the RNAprep pure kit (Tiangen, Beijing, China), and it was reverse-transcribed with a gDNA eraser according to the instructions of the Primescript RT reagent kit (Takara, Tokyo, Japan). The cDNA solution was used as a template for PCR amplification by specific primers (Supplementary Materials Table S1). Each 10 μL reaction mixture contained 5 μL of Ssofast EvaGreen Supermix and 0.5 μL of each gene-specific primer (500 nM final concentrations), 3.5 μL water, and 0.5 μL of the cDNA template. PCR amplifications were conducted by Bio-Rad CFX96 (Bio-Rad CFX Manager Software Version 1.6), followed by a PCR procedure with an initial denaturation at 95 °C for 30 s followed by 40 cycles of 95 °C for 5 s and 58 °C for 5 s. The *CpActin* and *CpTubulin* genes were used as the internal control for the expression analyses in wintersweet [32]; *Atactin* was used as the internal control for expression analyses in *Arabidopsis*. The comparative C_T_ method was used to quantify gene expression [41]. 

### 2.5. Plasmid Constructs and Plant Transformation

The full-length coding sequences of *CpCZF1* and *CpCZF2* were cloned into the modified plant binary vector pCAMBIA 2301G with the *35S* promoter at the *BamH*I and *SacI*, and the *EcoR*I and *BamH*I restriction sites, respectively, to generate the *35S*:*CpCZF1* and *35S*:*CpCZF2* constructs*.* The primers are listed in Supplementary Materials Table S1. The plant binary constructs were first transformed into the *Agrobacterium tumefaciens* strain GV3101 which were then transformed to *Arabidopsis* plants via the floral dip method. The transformants were selected on a MS medium with 50 mg/L Kanamycin. Homozygous T3 or T4 transgenic seedlings were used for the phenotypic investigation and the molecular assay.

### 2.6. Yeast Two-Hybrid Assay

The interaction between proteins was analyzed by the Matchmaker^®^ Gold Yeast Two-Hybrid System (Clontech, CA, USA). The full-length coding sequence of *CpCZF1* was cloned into a *pGBKT7 (BD*) vector at the *EcoR*I and *BamH*I sites to generate the *BD-CpCZF1* construct. Additionally, the coding sequence of *CpCZF2* was then cloned into the AD domain of the vector *pGADT7* to generate *pGADT7-CpCZF2* (*AD-CpCZF2*) at *BamH*I and *SaI*I sites. The constructs *BD-CpCZF1* and *AD-CpCZF2* were then co-transformed into the yeast strain Y2HGold according to the supplier’s instructions. The plasmids *AD-T*, *BD-53* provided by Clontech were co-transformed into the yeast strain Y2HGold to serve as the positive control; *BD-CpCZF1*, empty vector *AD*; *AD-CpCZF2*, empty vector *BD* and empty vector *AD*, empty vector *BD* were co-transformed, respectively, into the yeast strain Y2HGold to serve as negative controls. The co-transformed yeast cells were spread onto DDO/X plates to confirm the co-transformation efficiency. At the same time, the yeast cells were spread on selective medium QDO/X/A to assess the protein interactions of different pairs. The primers used are listed in Supplementary Materials Table S1.

### 2.7. BIFC Assay

The full-length coding sequence of *CpCZF1* was cloned into the vector *pSATNA-nEYFP-N1* that had an N-terminal fragment (aa 1–174) of yellow fluorescent protein (YFP) at the *Sai*I and *BamH*I sites; *CpCZF2* was cloned into the vector *pSATNA-cEYFP-N1* that had a C-terminal fragment (175–239) of YFP at the *Xho*I and *BamH*I sites. The constructs *CpCZF1-nEYFP* and *CpCZF2-cEYFP* were introduced into the *Agrobacterium tumefaciens* strain GV3101 and suspended to OD_600_ = 0.5, and were then mixed equally to infiltrate the lower epidermal cells of tobacco plants [42]. Specifically, if CpCZF1-nEYFP and CpCZF2-cEYFP were co-expressed in the lower epidermal cells of tobacco plants, a reconstituted fluorescence of YFP in the nucleus should be observed. After incubation in the dark at 22 °C for 36 h, a 2-mm × 2-mm area portion of the infiltrated leaves was cut out to observe the YFP under confocal microscopy (Olympus, FV-10-ASW, Tokyo, Japan). By following the BIFC control setting method of Horstman et al. [43], AtSZF1, a C3H-type zinc finger protein which is located in the nucleus [27], and belongs to the same subfamily as CpCZF1 and CpCZF2, was used as the partner of the negative control pairs. *AtSZF1* was cloned into the vector *pSATNA-cEYFP-N1*, *pSATNA-nEYFP-N1*, *pSATN-nEYFP-C1*, and *pSATN-cEYFP-C1* to generate the constructs of *AtSZF1-cEYFP*, *AtSZF1-nEYFP*, *nEYFP- AtSZF1*, and *cEYFP-AtSZF1*. *CpCZF1* was cloned into the vector *pSATNA-cEYFP-N1*, *pSATN-nEYFP-C1*, and *pSATN-cEYFP-C1* to generate *CpCZF1-cEYFP*, *nEYFP-CpCZF1*, and *cEYFP*-*CpCZF1.* The construct pairs *CpCZF1-nEYFP* and *AtSZF1-cEYFP*, *CpCZF1-nEYFP* and *cEYFP-AtSZF1*, *nEYFP-CpCZF1* and *AtSZF1-cEYFP*, *nEYFP-CpCZF1* and *cEYFP-AtSZF1*, *CpCZF1-cEYFP* and *AtSZF1-nEYFP*, *CpCZF1-cEYFP* and *nEYFP-AtSZF1*, *cEYFP*-*CpCZF1* and *AtSZF1-nEYFP*, and *cEYFP*-*CpCZF1* and *nEYFP-AtSZF1* were used as negative controls. The primers for these constructions are listed in Supplementary Materials Table S1.

## 3. Results

### 3.1. Isolation and Characterization of CpCZF1 and CpCZF2

**Figure 1:**
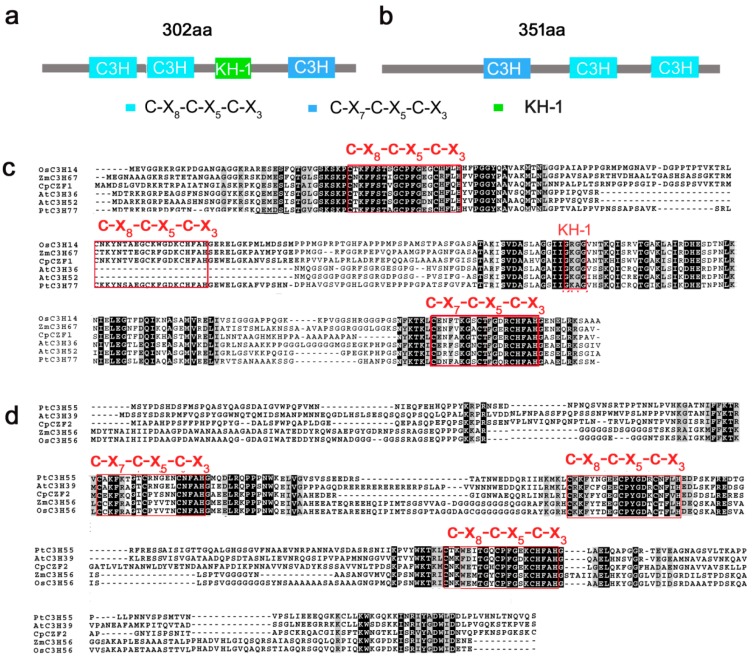
Sequence analysis of CpCZF1 and CpCZF2. Schematic diagrams of protein CpCZF1 (**a**) and CpCZF2 (**b**)*.* Multiple Sequence Alignment of CpCZF1 (**c**), CpCZF2 (**d**), and their orthologs from *Populus trichocarpa*, *Arabidopsis*, *Zea mays*, and *Oryza sativa*, respectively*.* The boxes in blue indicate the C-X_7_-C-X_5_-C-X_3_-H type motif; the boxes in sky-blue indicate the C-X_8_-C-X_5_-C-X_3_-H type motif; the green box indicates the KH-1 domain which is a RNA or single stranded DNA biding domain. The red box indicates the positions of the conserved domains.

Two C3H-type zinc finger protein genes (hereafter denominated as *CpCZF1* and *CpCZF2*) were isolated from flowers of wintersweet by using the RACE method. *CpCZF1* (Gene bank accession number: KY435926) contains a 909 bp open read frame (ORF) that encodes a peptide of 302 amino acid residues with a calculated molecular mass of 32.07 kDa and a theoretical isoelectric point of 9.35. Sequence analysis showed that three putative conserved motifs including two C-x_8_-C-x_5_-C-x_3_-H motifs on the N-terminal, one C-x_7_-C-x_5_-C-x_3_-H motif which is located in the C-terminal region of the peptide, and one RNA or single-stranded DNA binding KH-1 domain were detected (Figure 1a). *CpCZF2* (Gene bank accession number: KY435927) contains a 1056 bp ORF, which encodes a peptide of 351 amino acid residues with a calculated molecular mass of 39.19 kDa and a theoretical isoelectric point of 8.74. Sequence analysis showed that CpCZF2 contains one C-x_7_-C-x_5_-C-x_3_-H motif on the N-terminal region which is followed by two C-x_8_-C-x_5_-C-x_3_-H motifs (Figure 1b). 

**Figure 2:**
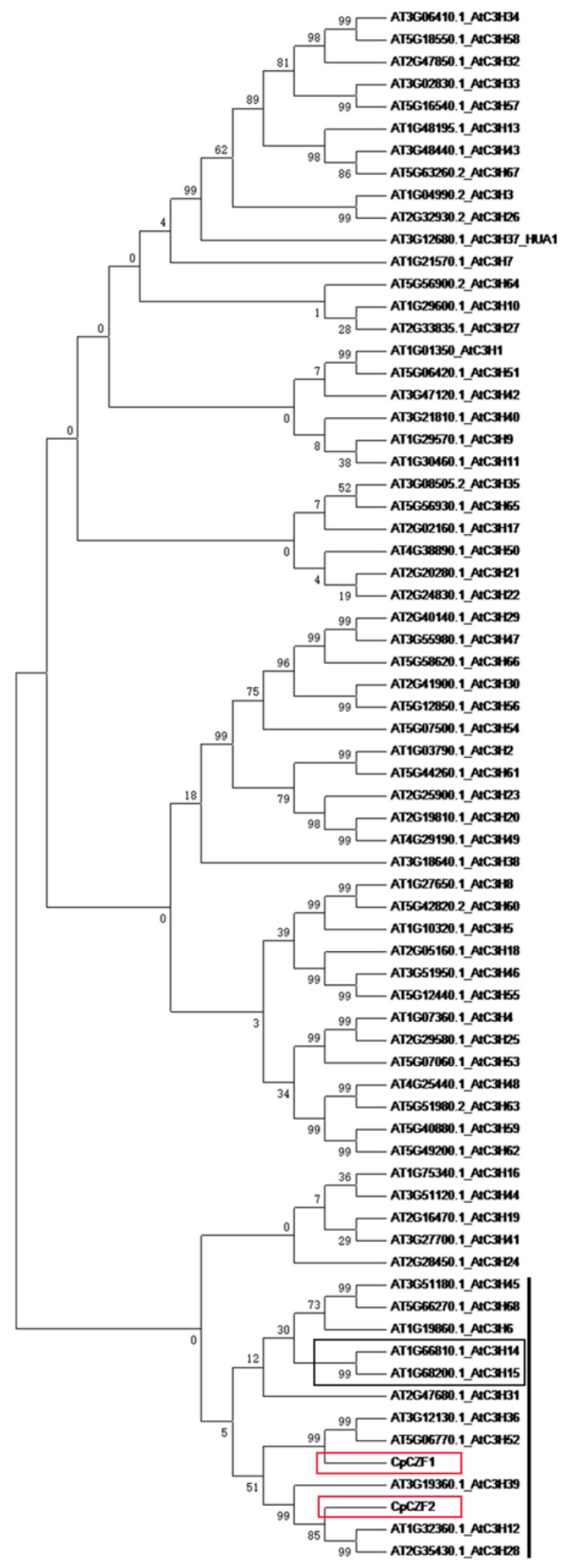
Phylogenetic analysis of CpCZF1, CpCZF2, and C3H-type proteins in *Arabidopsis*. CpCZF1, CpCZF2, and the accession numbers of zinc finger protein genes are shown in the phylogenetic tree. The blank line indicates the branch which CpCZF1 and CpCZF2 are clustered to. The red boxes indicate the position of CpCZF1 and CpCZF1; the blank box indicates the position of AtC3H14 and AtC3H15.

Alignment of the deduced amino acid showed that CpCZF1 and CpCZF2 share the same sequence structure and conserved motifs with their homologues from *Populus trichocarpa*, *Zea mays*, and *Oryza sativa* (Figure 1b–c). AtC3H36 and AtC3H52, the homologues of CpCZF1 from *Arabidopsis*, contain two CCCH motifs and one KH-1 domain, while CpCZF1 and other homologues contain three CCCH motifs and one KH-1 domain, but the data also showed that AtC3H36 and AtC3H52 (E-value = 9 × 10^−35^, identity = 50%; E-value = 7 × 10^−42^, identity= 46%, respectively) are highly homologous to CpCZF1 (Figure 1b). AtC3H39, the homologue of CpCZF2 from *Arabidopsis*, which shares the same number and type of CCCH motifs with *CpCZF2*, is highly homologous to CpCZF2 (E-value = 5 × 10^−49^, identity= 38%). Further analysis of the phylogenetic relationship between CpCZF1, CpCZF2, and the C3H-type proteins from *Arabidopsis* revealed that CpCZF1, CpCZF2, and 11 *Arabidopsis* C3H-type proteins were clustered together on a branch of the tree. AtC3H14 and AtC3H15, which function in anther development [28], were included in this branch. CpCZF1 has a close relation with AtC3H36 and AtC3H52, while CpCZF2 has a close relation with AtC3H39 (Figure 2).

### 3.2. CpCZF1 and CpCZF2 Located to Nucleus

**Figure 3:**
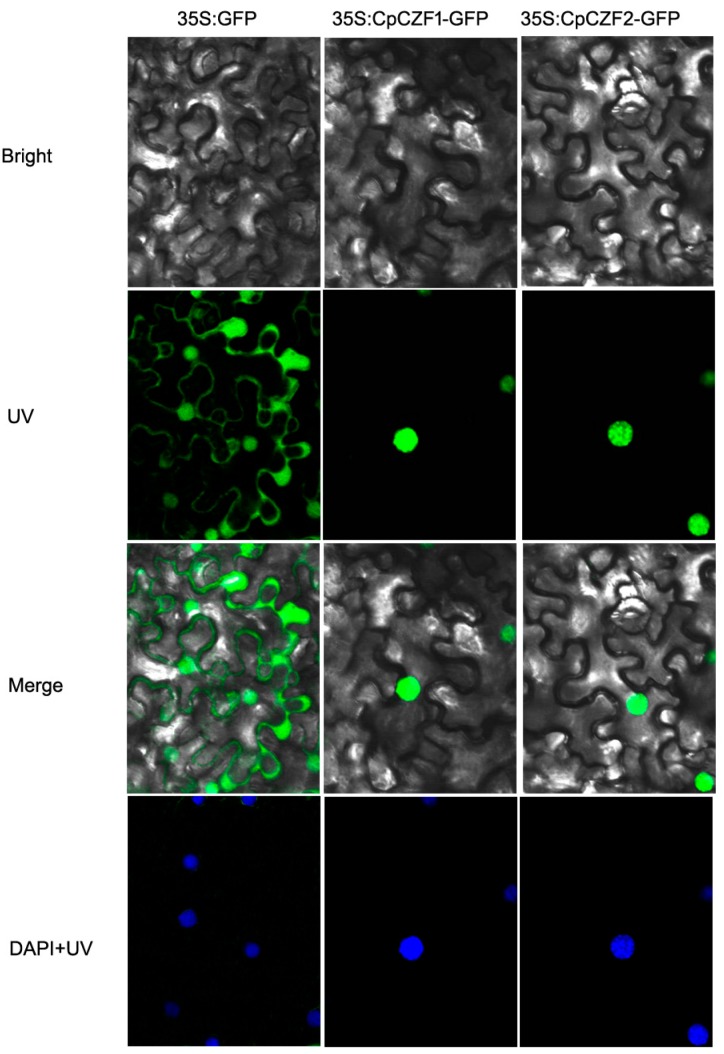
Subcellular localizations of CpCZF1 and CpCZF2 Tobacco leaf epidermis were transformed with *35S-GFP*, *35S:CpCZF1-GFP*, and *35S:CpCZF2-GFP* constructs by an *Agrobacterium* mediated infection. In this experiment, 35S-GFP was used as the control. The position of the nucleus was ensured by DAPI staining and bright-field images were compared.

To examine the subcellular localization of CpCZF1 and CpCZF2 in planta, a GFP (green fluorescent protein) reporter gene was fused in-frame to the C-terminus of *CpCZF1* and *CpCZF2* and transiently transformed into tobacco lower epidermal cells, separately. As shown in Figure 3, the GFP fluorescence of CpCZF1-GFP and CpCZF2-GFP was observed in the nucleus, whereas that of the control appeared in the cytoplasm and the nucleus. These results indicated that CpCZF1 and CpCZF2 are both nuclear-localized proteins and may function as transcriptional factors. 

### 3.3. The Spatiotemporal Expression Patterns of CpCZF1 and CpCZF2

**Figure 4:**
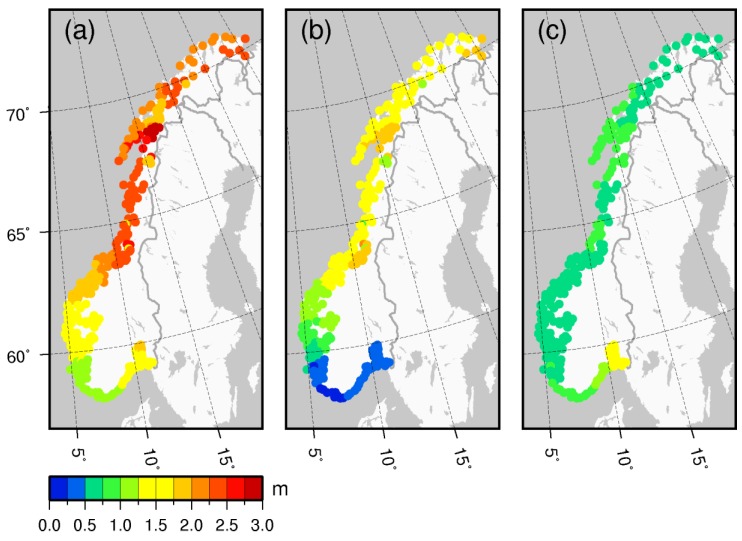
Expression patterns of *CpCZF1* and *CpCZF2* in different flower developmental stages. (**a**) Flower developmental stages. The transcriptional levels of *CpCZF1* (**b**) and *CpCZF2* (**d**) in flower buds (flowers) in the process of flower development. Relative expression of *CpCZF1* (**c**) and *CpCZF2* (**e**) in the tissues of wintersweet. SDS, sepal primordia differentiation stage; PDS, Petal primordia differentiation stage; PiDS, pistil primordia differentiation stage; StDS, stamen primordia differentiation stage; S1–S6, Stage1–Stage 6. Data were expressed relative to *CpActin* and *CpTublin* that served as the internal controls; the error bars represent the standard deviation per triplicate.

The qRT-PCR in flower buds (flowers) from all ten stages (Figure 4a) was performed to analyze the dynamic expression patterns of *CpCZF1* and *CpCZF2* in flower development. As shown in Figure 4b, the *CpCZF1* expression was at least 10-fold higher in flower buds at the floral primordia differentiation stages than in the flowers at the flowering stages; this was especially true for the petal primordial differentiation stage, which was approximately five-fold higher than expression at the sepal, stamen, and pistil primordia differentiation stages. After primordia differentiation, the transcripts of *CpCZF1* decreased dramatically. In addition, the transcript levels of *CpCZF2* were much higher in the three inner whorls’ primordial differentiation stages, especially in the petal primordial differentiation stage (Figure 4d). Moreover, the qRT-PCR was also performed for the root, stem, cotyledon, young leaves, mature leaves, and petals, stamens, and pistils from flowers of stage1 to analyze the expression patterns of *CpCZF1* and *CpCZF2* in different tissues of wintersweet. The *CpCZF1* expression level was extremely high in the flower organs, especially in the petals and stamens, but relatively low in the vegetative tissues (Figure 4c). The transcriptional level of *CpCZF2* in mature leaves was the highest among the vegetative tissues; however, the levels of petals and stamens were about 40-fold of those of mature leaves. Meanwhile, *CpCZF2* transcripts were also higher in flower organs than in vegetative tissues, especially for the stamens that had approximately 90-fold the expression level seen in mature leaves, which had the highest expression level among the vegetative tissues (Figure 4e). Unlike *CpCZF1*, which was highly expressed in both petals and stamens, *CpCZF2* was expressed more in stamens than in petals. These results showed that the expression patterns of *CpCZF1* and *CpCZF2* were similar, and were more abundantly expressed in flower organs and more highly expressed in primordial differentiation stages than in opening stages.

### 3.4. Effects of CpCZF1 and CpCZF2 Genes on Flower Organ Identities in Arabidopsis

**Figure 5:**
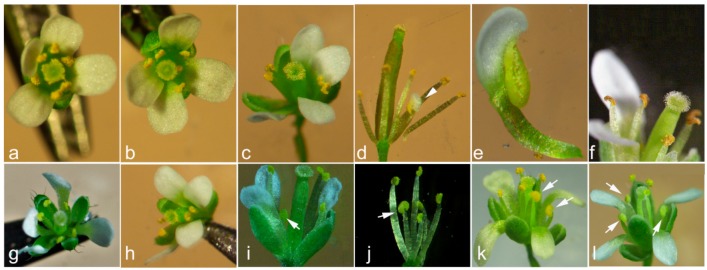
Phenotypic effects of *CpCZF1* and *CpCZF2* on transgenic *Arabidopsis.* (**a**) A WT flower with four long medial stamens and two short lateral stamens; (**b**–**f**) Flowers of 35S:CpCZF1 plants; (**b**) Flower with one lateral stamen lost; (**c**) Flower with both lateral stamens lost. (**d**) Flower with one lateral stamen changed to a partial petaloid; (**e**) An enlargement of a partial petaliod stamen; (**f**) Flower with anthers withered before the pistil could be fertilized; (**g**–**l**) Flowers of the 35S:CpCZF2 plants; (**g**) Flower with one lateral stamen lost; (**h**) Flower with both lateral stamens lost; (**i**) Flower with one staminode and one lateral stamen lost; (**j**) Flower with one staminode; (**k**) Flower with two staminodes; (**l**) Flower with three staminodes. Arrowhead indicates a partial petaloid stamen; arrows indicate the staminodes. To enhance the observations, the sepals and petals in photo (**d**), the sepals, petals, and carpels in photo (**j**) that were normal were all removed.

The functional roles of *CpCZF1* and *CpCZF2* were investigated in transgenic *Arabidopsis.* Eighteen *35S*:*CpCZF1* and 30 *35S*:*CpCZF2* transgenic lines obtained by Kanamycin selection were further confirmed as transgenic plants harboring *CpCZF1* and *CpCZF2* by PCR amplication (Supplementary Materials Figure S1a,b). As a typical cruciferous plant, *Arabidopsis* Col-0 flowers produce six stamens with two short lateral stamens and four medial stamens of identical length which are as long as the carpel at Stage 13 (Figure 5a) [10]. In our study, changes to the stamens in *35S*:*CpCZF1* and *35S*:*CpCZF2* transgenic plants were observed by comparing them to those of wild type (WT) organisms which were planted in the same condition as transgenic plants. 

In the 18 lines of *35S:CpCZF1* plants, four kinds of alterations were observed in transgenic flowers (Figure 5, Table 1): the loss of one (Figure 5b) or both lateral stamen(s) (Figure 5c), one partial petaliod lateral stamen (Figure 5d–e), and withering of the anthers occurred before the pistil could be fertilized (Figure 5f), and were observed in transgenic flowers. b, c, and f occurred in six lines (type1); b, c, d, and e were observed in 12 lines (type2). Statistical results of phenotype type 1 and type 2 showed that almost 30 out of 90 observed flowers lost one lateral stamen; approximately 12 flowers lost both lateral stamens; 11 flowers had a petaliod stamen and 12 had withered anthers. The ratio of flowers with abnormal stamens was 64% in phenotype type 1 and 59% in phenotype type 2 (Table 2).

In the 30 *35S:CpCZF2* transgenic lines, three kinds of alterations (Figure 5, Table 1): lost one lateral stamen (Figure 5g), lost both lateral stamens (Figure 5h), and stamen(s) changed to Staminode(s) (Figure 5i–l) which lost the capability of producing viable pollen, were observed on stamens in transgenic *Arabidopsis*, g and h were observed in nine lines (type 3), and g-l were observed in 21 lines (type 4). The statistical results in Table 2 showed that the number of abnormal flowers which lost one lateral stamen was 33 out of 90 flowers in both genotypes; the number of flowers for which both lateral stamen were lost was 12 in type 3 and 16 in type 4; the number of flowers which had staminode(s) was 13 out of 90. The ratio of flowers with abnormal stamens was 50% in type 3 and 69% in type 4.

The overexpression of *CpCZF1* and *CpCZF2* in *Arabidopsis* caused similar changes to the stamens. They both caused the reduction of lateral stamen(s), but also retained some difference.

### 3.5. Expression of the Stamen Identity-Related Genes 

**Figure 6:**
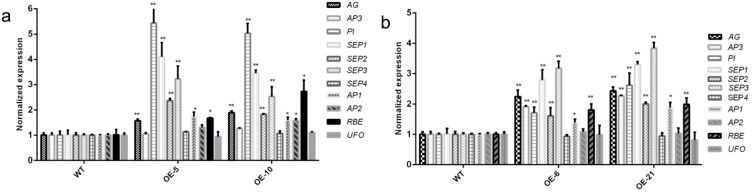
Effects of *CpCZF1* and *CpCZF2* overexpression on the transcript levels of regulatory genes related to stamen identity specification. Expression of stamen identity-related genes in the WT and the CpCZF1-OE (**a**) and CpCZF2-OE (**b**) flowers. The RNA was extracted from flowers at Stages 0–6 from the inflorescences of WT and transgenic *Arabidopsis*. Data were expressed relative to *AtActin*, which served as the internal control; the error bars represent the standard deviations per triplicate. The * and ** indicate a significant difference from WT at *p* < 0.05 and *p* < 0.01, respectively, as determined by the Student *t*-test.

To explore the possible causes of the changes in stamens of the overexpressed plants, the expression of the stamen identity genes, namely the B-function genes *AP3* and *PI*, the C-function gene *AG*, and the E-function gene *SEP1-4*, were analyzed in the WT and four types of the overexpressed plants. We also assayed the expression of the A-function genes, *APETALA1* (*AP1*) and *APETALA2* (*AP2*), and the flower organ boundary genes, *RABBIT EAR* (*RBE*) and *UNUSUAL FLORAL ORGANS* (*UFO*). As shown in Figure 6a, in the *35S*:*CpCZF1 Arabidopsis* inflorescence of lines OE-5 and OE-10, the transcripts of *PI* were approximately five-fold those of WT in both lines; *SEP1* and *SEP3* were approximately 3.5-fold; *AG* were approximately 1.5-fold; *SEP2* were approximately 1.7-fold; *AP1* were approximately 1.6-fold those of WT; and *AP2* were slightly upregulated. However, *AP3*, *SEP4*, and *UFO* showed no difference between the transgenic and WT plants. 

In the *35S*:*CpCZF2 Arabidopsis* inflorescence of lines OE-6 and OE-21, the transcripts of *AP1* were approximately 1.6-fold those of WT; *AP3*, *PI*, *AG*, *RBE*, and *SEP2* were all elevated by approximately two-fold over those of WT, while *SEP1* and *SEP3* were approximately three-fold higher than those of WT in the transgenic lines. Additionally, *SEP4*, *AP2*, and *UFO* showed no difference between the WT and transgenic plants (Figure 6b). 

### 3.6. Protein Interaction of CpCZF1 with CpCZF2 

**Figure 7:**
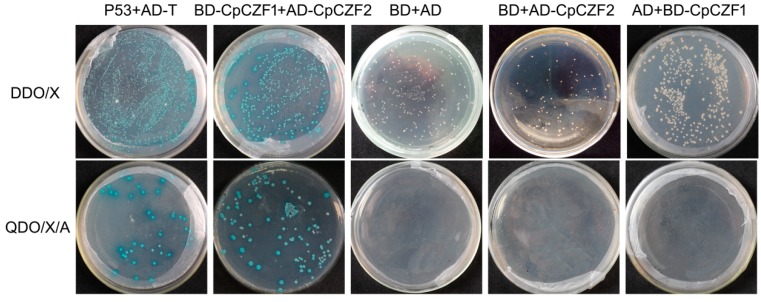
Yeast of two hybrids employed to detect the interactions of CpCZF1 and CpCZF2. Yeast cells co-transformed with *BD-CpCZF1* and *AD-CpCZF2* were spread onto selective media DDO/X and QDO/X/A to test the protein interactions. Yeast cells co-transformed with *pGBKT7-53* (*BD-53*) and *pGADT7-T* as the positive controls. Yeast cells co-transformed with *BD* and *AD*, *BD* and *AD-CpCZF2*, and *BD-CpCZF1* and *AD* as the negative controls. Selective media DDO/X was used to test the co-transformation efficiency; QDO/X/A media was used to test the positive interaction.

To investigate the potential relationship of CpCZF1 and CpCZF2 proteins, we tested the in vivo interaction by employing the yeast-two-hybrid system, and the result showed that the yeast cells harboring the testing pair and the positive control plasmid pair grew and turned blue on the DDO/X and QDO/X/A medium, respectively, which indicated that the testing pair, like the positive control, could activate the reporter genes resulting from the interaction of the two proteins. However, yeast cells harboring the negative control plasmid pairs, *AD-CpCZF2* and *BD*; *AD* and *BD-CpCZF1*; *AD* and *BD*, could grow on DDO/X medium but did not turn blue, which implied that both plasmids of each pair were co-transformed into yeast cells, but couldn’t activate the reporter gene; still, they couldn’t grow on QDO/X/A medium which meant that the proteins in each negative control pair couldn’t activate the reporter genes in the GAL4 system. These results indicate that CpCZF1 could interact with CpCZF2 in yeast (Figure 7). 

**Figure 8:**
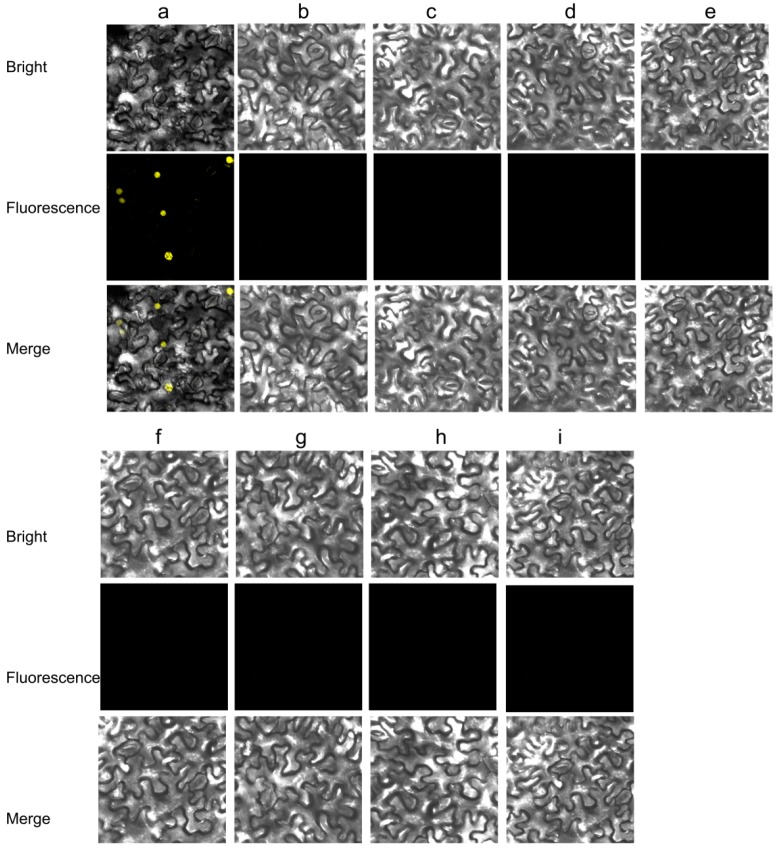
BIFC assays to confirm the interactions of CpCZF1 and CpCZF2. (**a**) the construct pair *CpCZF1-nEYFP* and *CpCZF2-cEYFP* was used to detect the protein interaction in tobacco cells. The construct pairs; (**b**–**i**) were used as negative controls; (**b**) *CpCZF1-nEYFP* and *AtSZF1-cEYFP*; (**d**) *nEYFP-CpCZF1* and *AtSZF1-cEYFP*; (**f**) *CpCZF1-cEYFP* and *AtSZF1-nEYFP*; (**c**) *CpCZF1-nEYFP* and *cEYFP-AtSZF1*; (**e**) *nEYFP-CpCZF1* and *cEYFP-AtSZF1*; (**g**) *CpCZF1-cEYFP* and *nEYFP-AtSZF1*; (**h**) *cEYFP*-*CpCZF1* and *AtSZF1-nEYFP*; (**i**) *cEYFP*-*CpCZF1* and *nEYFP-AtSZF1.*

An YFP BIFC system was employed to further confirm the interaction of CpCZF1 and CpCZF2 proteins in planta. The results showed that the YFP fluorescence was observed in the nucleus of the epidermal cells transfected with CpCZF1-nEYFP and CpCZF2-cEYFP, whereas no such fluorescence was detected in the cells transfected with the negative construct pairs (Figure 8). The reconstituted YFP was only observed in those cells with CpCZF1-nEYFP and CpCZF2-cEYFP, which indicates that CpCZF1 could interact with CpCZF2 in tobacco cells. 

## 4. Discussion

C3H-type zinc finger proteins are characterized by a zinc finger motif consisting of the residues of three cysteines and one histidine [18]. They exist in the wild in eukaryotic organisms, and a genome-wide analysis of the C3H-type zinc finger genes in the plant revealed that there are 68, 67, 68, 91, 80, and 34 C3H-type zinc finger genes in *Arabidopsis*, *Oryza sativa*, *Zea mays*, *Populus trichocarpa*, *Solanum lycopersicum*, and *Medicago truncatula*, respectively [20,44,45,46,47]. However, to date, very few of these genes have been functionally characterized. In this study, two C3H-type zinc finger protein genes which were differentially expressed in flower developmental stages [33] were isolated and functionally characterized in transgenic *Arabidopsis*. Both CpCZF1 and CpCZF2 have one C-X_7_-C-X_5_-C-X_3_-H motif and two C-X_8_-C-X_5_-C-X_3_-H motifs. These two kinds of motifs are the most common of the C3H-type zinc finger motifs. In *Arabidopsis*, 44/42 out of 68 C3H type proteins contain C-X_8_-C-X_5_-C-X_3_-H and C-X_7_-C-X_5_-C-X_3_-H motifs, while the number is 35/36 out of 67 in rice [44], and 32/29 out of 68 in corn, respectively [20]. In addition, CpCZF1 and CpCZF1 share a high sequence similarity with their orthologues from *Arabidopsis*, *Oryza sativa*, *Zea mays*, and *Populus trichocarpa*, respectively. Moreover, the phylogenetic analysis showed that CpCZF1 and CpCZF2 share a close relationship with AtC3H14 and AtC3H15, which were found to have a function in flower development [28]. Thus, CpCZF1 and CpCZF2 may share a similar biological function with their orthologues, possibly in flower development. 

The expression patterns of the C3H-type zinc finger protein genes show some consistency with their functions. *OsLIC* was highly expressed in collar, adaxial cells and tillering primordia, and the suppression of endogenous *OsLIC* expression resulted in drastically increased leaf and tiller angles, a shortened shoot height, and consequently reduced grain production in rice [21]. *PEI1* was specifically expressed throughout the embryo from the globular to late cotyledon stage, and further exploration revealed that *PEI1* plays an important role during *Arabidopsis* embryogenesis [48]. *AtTZF4* and *AtTZF5* were highly expressed in seeds and only at background levels in other tissues, and were found to be involved in seed germination [25]. *AtC3H14* and *AtC3H15* were expressed all over the plants, but were more highly expressed in the basal stems and anther, and have overlapping roles in second wall thickening and anther development [28]. *HUA1* RNA was detected throughout the plant, in the root, stem, leaves, and inflorescences, and was more abundant in inflorescences than in other tissues. Further studies also showed that *HUA1* RNA was detected in the inflorescence meristem, the inflorescence stem, and flowers of all stages; moreover, in the flower of each whorl, *HUA1* RNA was more concentrated in the petals, stamens, and carpels. Functional analysis uncovered that *HUA1* regulates stamen and carpel identities in the *AG* pathway in *Arabidopsis* [14]. In this study, *CpCZF1* and *CpCZF2* were expressed throughout the plant, but were more highly expressed in the flowers than in other tissues, and the expression pattern is similar to *HUA1* in *Arabidopsis.* Furthermore, higher transcripts levels of these two genes were found in the inner three whorls of the flower organs, which also share some similarity with *HUA1* [14]. In addition, *CpCZF1* and *CpCZF2* were also more highly expressed in primordia differentiation stages than in the opening stages. All the results suggest that these two genes may function in flower organ development. 

Plant C3H-type zinc finger proteins are involved in multiple developmental processes, including embryo development [48], secondary xylem formation [49], seed germination [25], leaf senescence [22,23], and flower development [14,15,28,31]. In this study, *CpCZF1* and *CpCZF2*, both caused alterations on the stamens when overexpressed in the *Arabidopsis* plant, respectively, which indicated that they may function in regulating flower development, especially stamen identity specification in transgenic *Arabidopsis*. Furthermore, the overexpression of *CpCZF1* and *CpCZF2* in *Arabidopsis* caused a reduction of the lateral stamen. Nonetheless, they also caused different alterations, i.e., the transition of one lateral stamen to a partial petaloid; the stamens and carpels that developed out of sync only occurred in the *35S*:*CpCZF1* plants, whereas staminodes were only observed in the *35S*:*CpCZF2* plants. These results indicate that *CpCZF1* and *CpCZF2* have the same function in stamen identity, yet also retain noteworthy differences. 

According to the floral quartet model, the identity of the floral organs is specified by quaternary protein complexes composed of A-, B-, C-, D-, and E-function proteins [50,51]. It suggested that the stamen is specified by the quartet of PI-AP3-SEP-AG. Moreover, the relative expression levels of the A-function genes, which were repressors of *AG*, could also affect the stamen identity via affecting the expression of *AG*. Therefore, the expression level of *AG*, *SEP* genes, *AP3*, *PI*, *AP1*, and *AP2* were analyzed in the inflorescence of WT and transgenic lines to investigate the possible causes of the stamen development disruption in transgenic plants. In addition, considering the petaliod stamen in the transgenic lines, the floral organ boundary genes, such as *RBE* and *UFO*, which play roles in petal development [52,53,54], were also assayed. As shown in Figure 6a, the overexpression of *CpCZF1* in *Arabidopsis* caused the elevation of *PI*, *AG*, *SEP1*-*3*, *AP1*, *AP2*, and *RBE*—especially *PI*, *SEP1*, *SEP3*, and *RBE—*which suggests that *CpCZF1* may mainly act as a regulator of the *PI*, *SEP*, and *RBE* genes. The overexpression of *CpCZF2* in *Arabidopsis* up-regulated the expression level of all the genes analyzed except *SEP4*, *AP2*, and *UFO*, but especially that of the E-function genes *SEP1* and *SEP3* (Figure 6b). The qRT-PCR results showed that the overexpression of *CpCZF1* and *CpCZF2* disturbed the expression of the stamen identity-related genes. The abnormal stamens might not be directly caused by the upregulation of one or two of the stamen identity-related genes. It’s probably that the expression disturbance of a series of the related genes may affect the formation of the quaternary protein complexes which thereafter results in the abnormal stamens in transgenic plants. The abnormal flower organs and disturbed expression of stamen identity-related genes showed some similarities to other genes. The overexpression of *HoAGL6* in *Arabidopsis* caused abnormal flowerer organs including reduced stamen numbers, and gene expression analysis revealed that the expression of *AG* and *SEP* was significantly elevated in transgenic plants [55]. The overexpression of *CpAGL6* caused abnormal stamens in *Arabidopsis* plants, and the expression of *PI*, *AP3*, *AG*, and *SEP* genes was upregulated [36]. Additionally, the Ectopic expression of *PvMADS56* in *Arabidopsis* caused abnormal organs and the loss of all stamens, and the expression of *AP1*, *AP3*, *PI*, and *AG* was downregulated [56]. However, the up-regulation of these genes in *OE-CpCZF1* and *OE-CpCZF2* plants was very different from the situation in *hua-1* mutations in which the *AG* RNA was not affected [14]. These differences imply that *CpCZF1*, *CpCZF2*, and *HUA1* may affect the stamen identity specification by different ways. 

The results of the yeast-two-hybrid assays and the BIFC experiment demonstrated that the interaction of CpCZF1 and CpCZF2 could not only be observed in yeast, but also visualized in tobacco cells. This interaction, to some extent, could give a possible explanation for the similar function of these two genes. This is not rare for interaction proteins which have similar functions. wrky18, wrky40, and wrky60 are three transcriptional factors that can interact with each other, and play an overlapping role in plant responses to different types of microbial pathogens [57]. 

In conclusion, our results demonstrate that CpCZF1 and CpCZF2 are two C3H-type zinc finger proteins which are located in the nucleus and which can interact with each other in vivo and in plant cells. These two genes are highly expressed in the flower organs and in the petal and stamen primordia differentiation stages of wintersweet. Additionally, both genes caused abnormal stamen in transgenic *Arabidopsis* which likely function in regulating stamen identity-related genes. The functional characterization of *CpCZF1* and *CpCZF2* could broaden our understanding of the roles that C3H-type zinc finger genes play in plants, especially the reproductive roles played by their orthologs; these findings also lend supplementary evidence for C3H-type zinc finger proteins in regulating flower development, thereby expanding our understanding of flower development in wintersweet, and possibly in other woody plants. To our knowledge, CpCZF1 and CpCZF2 were the first two C3H-type zinc finger proteins to be identified and functionally characterized in wintersweet, even in other species. Hence, more studies exploring the function of other orthologues need to be carried out.

## Figures and Tables

**Table 1:** Phenotypical characterization of flower morphology in *35S:CpCZF1* and *35S:CpCZF2* transgenic *Arabidopsis.*

Genotype	No. of Transgenic Lines	Phenotype
*35S:CpCZF1*	6	stamen number reduced and/or anthers withered before the pistil could be fertilized (type 1: Figure 5b,c,f)
	12	stamen number reduced and /or partial petaliod lateral stamen (type 2: Figure 5b–e)
*35S:CpCZF2*	9	stamen number reduced (type 3: Figure 5g,h)
	21	stamen number reduced and /or stamen(s) changed to staminode (s) (type 4: Figure 5g–i)

**Table 2:** Statistical results of the abnormal stamens in wild type (WT) and overexpression plants.

Genotype	Type	1–30 ^a^/90 ^b^					
		Lost 1 Stamen	Lost 2 Stamens	Petaliod	Withered Anther	Staminode(s)	Ratio
WT		4	0	0	0	0	0.04
*35S:CpCZF1*	1	32	14	0	12	0	0.64
	2	30	12	11	0	0	0.59
*35S:CpCZF2*	3	33	12	0	0	0	0.5
	4	33	16	0	0	13	0.69

**^a^** Position of flowers on the inflorescence; ^b^ Total number of flowers counted.

## Supplementary Materials

The following are available online at www.mdpi.com/2073-4425/8/8/199/s1. Table S1: List of the DNA primers used in this study, Figure S1: PCR amplication to detect the transgenic *Arabidopsis* lines.

## Abbreviations

The following abbreviations are used in this manuscript:

C3H	Cysteine_3_Histidine
DDO/X	SD/-Trp/-Leu/X-α-Gal
QDO/X/A	SD/-Trp/-Leu/-His/-Ade/X-α-Gal/Aureobasidin
qRT-PCR	Quantitative reverse transcriptase–polymerase chain reaction
Y2H	Yeast two hybrid
WT	Wild type
OE	Overexpression
