# Supplementary material for: Two C3H Type Zinc Finger Protein Genes, CpCZF1 and CpCZF2, from Chimonanthus praecox Affect Stamen Development in Arabidopsis
Source: Genes (Basel). 2017 Aug 10;8(8):199. doi: 10.3390/genes8080199 (PMC5575663; doi:10.3390/genes8080199)
Supplement: Supplementary file 1 [file genes-08-00199-s001.pdf]

Supplementary Table 1 List of the DNA Primers Used in This Study

name	5'-3' sequence	usage
V-CpCZF1-R	GgaattcCTCTAAGATTAACTCAAACTGCTGGCT	plant expression vector
V-CpCZF1-F	CggatccATGGCAATGGATTCTTTGGGTGTG	
V-CpCZF2-F	CggatccATGATTGCTCCTGCTCATCCACCAT	
V-CpCZF2-R	GgagctcAACTCAGCATTTTGATTTGCCAGGG	
L-CpCZF2-F:	gagctcATGATTGCTCCTGCTCATCCACCAT	Sublocalizition
L-CpCZF2-R:	ggatccGCATTTTGATTTGCCAGGGGAG	
L-CpCZF1-F	ggatccATGGCAATGGATTCTTTGGGTGTG	
L-CpCZF1-R	gtcgacAACTGCTGGCTTGCGCAACTCACT	
Y-CpCZF1-F	gaattcATGGCAATGGATTCTTTGGGTGTGG	**Y2**H
Y-CpCZF1-R	ggatccTCAAACTGCTGGCTTGCGC	
Y-CpCZF2-F:	ggatccAAATGATTGCTCCTGCTCATCCACCAT	
Y-CpCZF2-R:	gtcgacTCAGCATTTTGATTTGCCAGGG	
B-CpCZF1-F	gtcgacATGGCAATGGATTCTTTGGGTGTG	BIFC
B-CpCZF1-R	ggatccAACTGCTGGCTTGCGCAACTCACT	
B-CpCZF2-F:	CctcgagATGATTGCTCCTGCTCATCCACCAT	
B-CpCZF2-R:	CggatccGCATTTTGATTTGCCAGGGGAG	
B-AtSZF-F	CctcgagATGTGCAGTGGACCAAAGAGCAATC	
B-AtSZF-R	ggatccCACCACAGTCTGCTCCTTCTCTCTGT	
CpActin-Q-F	GTTATGGTTGGGATGGGACAGAAAG	qRT-PCR
CpActin-Q-R	GGGCTTCAGTAAGGAAACAGGA	
CpTublin-Q-F	TAGTGACAAGACAGTAGGTGGAGGT	
CpTublin-Q-F	GTAGGTTCCAGTCCTCACTTCATC	
AtActin-Q-F	CTTCGTCTTCCACTTCAG	
AtActin-Q-R	ATCATACCAGTCTCAACAC	
AtAG-Q-F	TGCAACCTAACAATCACCATTACTC	
AtAG-Q-R	ACCCAATTCTGGTTTTTATTCACTC	
AtPI-Q-F	CCAAATCTTCAGGAAAAGATTAT	
AtPI-Q-R	AAGACAAACTAAAGACCACGATATT	
AtAP3-Q-F	CTCTGCCTCTGACATCATTACCTTC	
AtAP3-Q-R	GTTTTAGCAACACCATGCCTTATG	
AtSEP1-Q-F	TGTTACAGCATTGGTTGGTGTTCT	
AtSEP1-Q-R	ATGATCCTCGGCAGCATCAC	
AtSEP2-Q-F	CTGTTCTACGATACCAAGCCTAGTG	
AtSEP2-Q-R	AAGCACCATGAAGAAGAGCAAACTA	
AtSEP3-Q-F	CCAACTCTATTTGAATCTTTCTCAC	
AtSEP3-Q-R	ACAAGACAGAAAACATGAGAGAGGT	
AtSEP4-Q-F	ACTCTGCAACAACATCACAGAATGT	
AtSEP4-Q-R	CAAAAATCAAATGACACAAGCAATG	
CpCZF1-Q-F:	TCCAACTGGTGTAGGAAGCAAATCG	
CpCZF1-Q-R:	GATTGTTCAGATTGGTCATTTGGGC	
CpCZF2-Q-F:	GAGGCAAGCATGTGGTATAAAGAGC	
CpCZF2-Q-R:	TCAATCCAATCAGCATAAACACGG	
CpCZF1-RB0	GTACTGTTGGATGCCCATTCGGTG	5’RACE
CpCZF1-RB2	AATGGCAGCCTTCACCGAATGGG	
CpCZF2-RB2	CTGAGGCCAGAACGACAACGCATC	
CpCZF2-RB0	AGCACTCTGGTGTTCAAGGTTGGGT	

Letters in lower case indicate the restriction enzyme sites

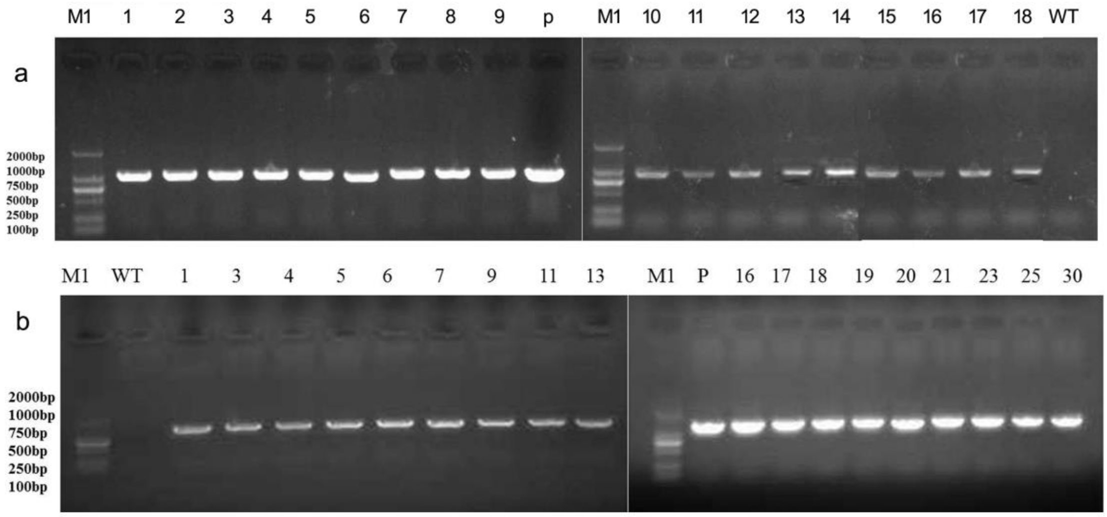

Supplementary Fig. S1 PCR amplication to detect the transgenic *Arabidopsis* lines

(a) PCR amplication to detect the *CpCZF1* gene in DNA of 18 transgenic Arabidopsis lines; (b) PCR amplication to detect the *CpCZF2* gene in DNA of 18 randomly selected transgenic Arabidopsis lines. M1: DNA Marker DL2000;1-30: transgenic Arabidopsis line 1-line18; P: positive control (pCAMBIA2301G- CpCZF1); WT: negative control (Wild Type)
